# How Big Is Your Y? A Genome Sequence-Based Estimate of the Size of the Male-Specific Region in *Megaselia scalaris*

**DOI:** 10.1534/g3.114.015057

**Published:** 2014-11-07

**Authors:** Kenneth B. Hoehn, Mohamed A. F. Noor

**Affiliations:** * Department of Zoology, University of Oxford, Oxford OX1 3PS, United Kingdom; † Biology Department, Duke University, Durham, North Carolina 27708

**Keywords:** sex chromosome evolution, neo-Y chromosome, male-specific, YGS

## Abstract

The scuttle fly, *Megaselia scalaris*, is often cited as a model in which to study early sex chromosome evolution because of its homomorphic sex chromosomes, low but measurable molecular differentiation between sex chromosomes, and occasional transposition of the male-determining element to different chromosomes in laboratory cultures. Counterintuitively, natural isolates consistently show sex linkage to the second chromosome. Frequent natural transposition of the male-determining element should lead to the loss of male specificity of any nontransposed material on the previous sex-linked chromosome pair. Using next-generation sequencing data from a newly obtained natural isolate of *M. scalaris*, we show that even highly conservative estimates for the size of the male-specific genome are likely too large to be contained within a transposable element. This result strongly suggests that transposition of the male-determining region either is extremely rare or has not persisted recently in natural populations, allowing for differentiation of the sex chromosomes of this species.

Theories of sex chromosome evolution suggest that it begins when a cluster of sex-determining genes evolves on the same chromosome as genes conferring sex-specific fitness effects (Charlesworth and Charlesworth 1978; Bull 1983; Charlesworth *et al.* 2005; Ellegren 2011). Selection favors reduced recombination among these genes, and sexually antagonistic functions accrue in the linked region, causing the autosomal pair to diverge into proto-X or Y chromosomes. The proto-Y region eventually only exists in the heterozygous form, and the absence of recombination causes it to accumulate mutations and diverge from the proto-X region. The nonrecombining region grows over time, creating “evolutionary strata” (Lahn and Page 1999) of divergence between the proto-X and Y chromosomes, and may eventually result in two chromosomes that essentially do not exchange alleles. Testing theories of sex chromosome evolution requires studying proto-X and Y chromosome at different (especially early) stages along this process, such as found in members of the genus Silene (Bergero *et al.* 2007; Mrackova *et al.* 2008). However, understanding the very earliest stages necessitates studying the origin of a new sex-determining region immediately after it arises.

The scuttle fly, *Megaselia scalaris*, has been proposed as a model system for the earliest steps of sex chromosome evolution (Traut 1994). This species sports three chromosome pairs, but none of the mitotic metaphase chromosomes appear heteromorphic via microscopy (Traut 2010). Nonetheless, some X-Y chromosome differentiation was identified via Southern hybridization (Willhoeft and Traut 1990), RAPD markers (Traut 1994), and DNA sequencing of a 1.8-kb segment (Traut and Wollert 1998). More strikingly, based on linkage to phenotypic markers, the male-determining region transposes among the chromosomes in some laboratory cultures at a detectable rate of 0.05%–0.4% (Mainx 1964; Willhoeft and Traut 1990; Traut 1994; Traut 2010). In principle, transposition of a male-determining region off the proto-Y would allow the remainder of the proto-Y sequence variants to spread into females, and thus eliminate their male specificity. Nonetheless, despite the detectably frequent transposition observed in laboratory culture, natural isolates from worldwide collections all have the male-determining region associated with the same chromosome (Traut 2010).

These findings raise the question of whether *Megaselia scalaris* has a large, distinct male-specific region (a true “Y-chromosome”), or whether transposition by the nonrecombining male-determining region occurs at a detectable rate in natural populations and the male-specific region is, in fact, quite small (perhaps encompassing only the transposing fragment). Willhoeft and Traut (1990) identified several male-specific bands in Southern hybridizations of *M. scalaris* DNA, suggesting the male-specific region may be large. In particular, they found that one of eight randomly chosen DNA probes gave a purely male-specific band in all strains studied. Assuming these probes are independent of each other, this observation suggests a male-specific genome size of 12.5%. However, given that only eight probes were used, this is far from a conclusive result.

In this study, we analyze genome sequences from male and female *Megaselia scalaris* to estimate the size of the male-specific region in this species. If the male-specific region is very large, then frequent transposition of the male-determining region and establishment on new chromosomes is improbable in natural populations. Taking the most conservative method possible, we find that the male-specific genome is likely too large to be located on a small, frequently transposing element. However, the male-specific genome could still represent a fraction of an otherwise large homomorphic sex chromosome.

## Materials and Methods

The source strain was derived from a single wild-caught, fertilized female (“Durham, NC 2”). This strain was initially inbred for five to seven generations using single full-sibling pairs, and then maintained as a stock in the laboratory for 1 yr. Prior to sample collection, two further rounds of inbreeding were conducted using one single full-sibling pair per round. Sequences were obtained from 30–50 adult males and females separately. For sequencing the females, we used four lanes of Illumina/Solexa GA, paired-end, 75-bp reads, giving a total of 79,232,896 sequences and 5.9×10^9^ bp. The insert lengths for these paired-end Illumina reads are expected to be approximately 300 bp. For the males, we used also used four lanes of Illumina/Solexa GA, nonpaired-end, 76-bp reads, giving a total of 111,268,292 sequences and 8.4×10^9^ bp. Using an estimated haploid genome size of 500 Mb (Rasmussen and Noor 2009), this gives a coverage of 5.9×10^9^/5×10^8^ ∼12× coverage for females and 8.4×10^9^/5×10^8^ ∼17× coverage for males. The sequencing reads from males were assembled using SOAPdenovo2 (k =42, N50 = 292) (Luo *et al.* 2012). Statistics for the male assembly are available in Supporting Information, Table S1.

We utilized Y Genome Scan (YGS), a kmer count-based method (Carvalho and Clark 2013) to determine which of these contigs were potentially male-specific. This method scans across each contig from a male assembly, storing each unmatched single copy (USC) kmer in each contig and determining if there are any matching kmers in female short read data. From this, plotting the size of each contig against the percentage of USC kmers that are unmatched in the female short read data usually gives two distinct peaks at 100% USC kmers and 0% USC kmers. These peaks are inferred to be contigs from the Y and autosomal chromosomes, respectively. In our case, we filtered our female short reads using Jellyfish (Marçais and Kingsford 2011) (m = 15, minquality = 20, quality-start = 33, lower-count = 5). We ran YGS, as recommended, with 15 mers.

We used a Bayesian approach for size inference of the male-specific genome. Because both our male and female assemblies were low-coverage—which we expect to artificially inflate the number of USC kmers, and thus artificially inflate the number male-specific contigs—we took the most conservative approach available and only considered contigs with 100% USC kmers as putatively male-specific. We further modeled the probability that a putatively male-specific contig was actually male-specific as a binomial likelihood parameterized by the number of USC and matched single copy (MSC) kmers. Assuming a beta(a,b) prior, the posterior probability of a putatively male-specific contig being male-specific is a beta distribution:

$$P\left(\mathrm{Male specific contig}|\mathrm{kmers}\right)=\frac{\text{1}}{B\left(a,b\right)}{\left(X\right)}^{a+\mathrm{USC kmers}-1}{\left(\text{1}-X\right)}^{b+\mathrm{MSC kmers}-1}$$

where a / (a + b) = 2×10^−4^, giving a prior expected male-specific genome size of 0.02%. The sum a + b is a measure of how heavily the prior is weighted relative to the data. Given a prior weight (a + b = PW), this system of equations solves to obtain a unique set of prior parameters by the formulas: a = PW * 2×10^−4^ and b = PW * (1 − 2×10^−4^).

The expected value of this distribution is then

$$E\left(P\left(\mathrm{Male specific contig}|\mathrm{kmers}\right)\right)=\frac{a+\mathrm{USC kmers}}{a+b+\mathrm{USC kmers}+\mathrm{MC kmers}}$$

Choosing a prior weight of 1 means that a 100% USC kmer contig with only one SC kmer has an approximately 50% probability of being male-specific. A prior weight of 22—the median SC kmer count across all contigs in our *Megaselia* assembly—means that 100% USC kmer contigs with 22 SC kmers have an approximately 50% probability of being male-specific. Our process for male-specific genome size estimation is—using different contig size cutoffs and prior weights—to calculate this expected value for each contig and multiply by the length of the contig to get a kmer-weighted estimate of the size of the male-specific genome. Specifically:

1. Set prior sum a + b, and then solve for a / (a + b) = 2×10^−4^.
2. Remove all contigs smaller than a selected size cutoff.
3. For each contig of 100% USC kmers, calculate the expected probability for male specificity using posterior distribution, multiply this probability by the length of the contig, and sum across all such contigs.
4. Divide the sum from (3) by the total length of all contigs above the size cutoff, giving the expected male-specific proportion of the genome.
5. Repeat 1–4 with different size cutoffs and prior weights.

There are two boundary cases for what is meant by “expected male-specific proportion of the genome” (hereafter MSP). In the first case, if there is very low genetic diversity in the individual sequenced—such as from a highly inbred line—we expect each locus in the genome to be represented by no more than one contig and, thus,

$${\mathrm{MSP}}_{d}=\frac{Y}{X+Y+\mathrm{Autosomes}}$$

which is approximately the MSP of the haploid genome. In the opposite most extreme case, if parental autosomes are very divergent in the individual sequenced, then we expect small, diverse contigs covering the same locus to not assemble together, meaning each locus will be represented by two contigs. Thus,

$${\mathrm{MSP}}_{d}=\frac{Y}{X+Y+\text{2}*\mathrm{Autosomes}}$$

which is the MSP of the diploid genome. MSP_d_ is the more immediately usable and interpretable. To give the most conservative lower bound for MSP_d_, we assume that the MSP we measure from step 4 is MSP_h_ and then divide by two to obtain a lower bound for MSP_d_. From there, we can give a lower bound for the absolute size of the male-specific genome by multiplying MSP_d_ by the diploid genome size.

To test this technique, we also tried it using a low coverage female genome assembly tested under Carvalho and Clark (2013). However, in this case the male-specific peak was shifted below 100%, and as such we considered contigs with >80% USC kmers.

## Results

Our application of the YGS method to locating putatively male-specific contigs showed what we interpret as a successful separation of male-specific and autosomal contigs. A heatmap (Figure S1) plotting log(contig size) *vs.* percent USC kmers shows two clear peaks at 0% USC kmers and 100% USC kmers. Like Carvalho and Clark (2013), we interpret these to be the result of male-specific contigs not matching female short read kmers. Because we anticipate low coverage assemblies to bias results toward higher percentages of USC kmers, we take the most conservative approach and only consider contigs with 100% USC kmers as putatively male specific.

We applied the procedure of estimating mal-specific genome size using contig size cutoffs of 0 bp, 1000 bp, 2000 bp, and 3000 bp. We further used different prior weights (a + b) between 1 and 70. This altered how heavily we weighted the expectation of small (0.02%) male genome size. From these results, we obtained a generally consistent estimation of the MSP for *M*. *scalaris*.

A prior weight of 1 gives a size estimate between 9.9% and 5.3%. Increasing the prior weight to 22—the median SC kmer count across all contigs—lowered this to between 3.5% and 2.4%. Increasing the prior rates further caused a convergence of MSP, the lowest estimation was at a prior weight of 70, which returned between 1.5% and 1.2% MSP. A prior weight of 70 is likely far too conservative. These results are summarized in Figure S2. Taking these results, we divide the percentage by two to be sure the estimate is below the MSP for the diploid genome (MSP_d_) and multiply by the diploid genome size of ∼1000 Mb (Rasmussen and Noor 2009). From this, we estimate a lower bound MSP_d_ for the prior weight of 1 as 26.5 Mb, 12 Mb for the median prior weight, and 5.5 Mb for the highly conservative weight of 70.

As a control, we applied this method to *Drosophila virilis* as well (also used in the Carvalho and Clark 2013 study), which differed from our *Megaselia* data set in that it had significantly higher coverage in its male assembly and substantially fewer contigs. Furthermore, in this case there was a distinct peak of % USC kmers not at 100% but between 80% and 100%, so we considered all contigs in this range to be putatively male-specific. In this analysis, we applied size cutoffs between 0 and 10 kb and, because the contigs had a kmer count median of 259 and a 90^th^ percentile of 4971, we applied prior weights between 1 and 10,000. These all gave a steady lower bound estimate of 6.6% for the least conservative, 6.1% for the median, and 3.6% for the most conservative. These results are summarized in Figure S3.

Flow cytometry studies (Gregory and Johnston 2008) suggest that the Y chromosome of *D. virilis* is approximately as large as the X, and that the X is approximately as large as each of the four autosomes (FlyBase: http://flybase.org/reports/FBsp00000251.html). This gives an approximate expected MSP_d_ of 1/10 = 0.1, and MSP_h_ of 1/6 = 0.167. The technique clearly acts, as we anticipated, as a very conservative lower bound. Because the male assembly for *D. virilis* is much more complete than *Megaselia*, it makes sense that the least conservative estimates of MSP would be the most accurate estimation because we anticipate far fewer false-positive scaffolds. In *Megaselia*, we expect a large portion of our 100% USC contigs, particularly those of small size, to be false positives, so a more conservative approach is warranted. However, the MSP of *Megaselia* may still be much larger than estimated through this conservative method. Further, because our assembly is at such a lower coverage, we expect the MSP measured through our method to be closer to MSP_d_ than to MSP_h_, which, if true, would effectively double our estimates of the lower bound size of the male-specific genome of *Megaselia*.

## Discussion

*Megaselia scalaris* has long been cited as an example of early sex chromosome evolution. Early karyotypic studies of this fly’s chromosomes revealed homomorphic sex chromosomes (Traut *et al.* 1990), and molecular studies revealed some differentiation between the two chromosomes (Willhoeft and Traut 1990). However, Willhoeft and Traut (1990) showed that the male determining region (M) regularly transposes from the second chromosome to the third in the laboratory. Follow-up studies on the same lines (Traut 1994) suggested that this transfer did not occur as a translocation at the chromosome ends, but as a comparatively small, complex transposable element.

The frequent transposition of the male determining region is seemingly at odds with observations that natural isolates appear to always show sex linkage to the second chromosome (Traut 2010). Frequent and persistent transposition in natural populations would rapidly lead to the decay of male specificity of previously male-specific parts of the genome. Hence, if transposition were frequent, then we would expect the size of the male-specific region to be not much larger than the size of known transposable elements. However, all of our estimates of the lower bound size of the male-specific genome—even those taken from a highly conservative approach—are multiple orders of magnitude larger than the lengths of known transposable elements, the largest of which, “Mavericks,” are on the order of 20–50 kb (Feschotte and Pritham 2007). Our findings indicate that location of the male-determining region in this natural isolate (Durham 2) has been persistent long enough in natural populations for a large male-specific genomic region to evolve. However, these results do not necessarily conflict with observations of transposition in the laboratory and may be reconciled through population cage experiments (Traut 2010) showing sex linkage to the second chromosome gives a selective advantage in well-aerated cages, but is lost under crowded cage conditions. Hence, more generally, these findings could suggest that the male-determining region is able to transpose in natural populations, but that it is rapidly selected against when linked to the first or third chromosome.

Ultimately, although our results suggest that transposition of the male-determining element of *Megaselia scalaris* has not occurred or persisted recently in natural populations, our results are not wholly inconsistent with the hypothesis that *Megaselia scalaris* is currently at an early stage of sex chromosome evolution. The full size of the male-specific region and an accurate estimate of the age of the transposition event that established it are beyond the scope of this analysis. However, we were able to show that a highly conservative estimate of the size of the male-specific genome reveals that it is far too large to be contained within a known transposable element, indicating that the male-determining element has remained linked to its current position long enough for a male-specific genome of at least several million bases to be established.

## Supplementary Material

Supporting Information

## References

[bib1] Bergero R. Forrest A. Kamau E. Charlesworth D., 2007 Evolutionary strata on the X chromosomes of the Dioecious plant Silene latifolia: Evidence from new sex-linked genes. Genetics 175: 1945–1954. 17287532 10.1534/genetics.106.070110 PMC1855140

[bib2] Bull J. J., 1983 Evolution of Sex Determining Mechanisms. Benjamin/Cummings Pub. Co., Menlo Park, California.

[bib3] Carvalho A. B. Clark A. G., 2013 Efficient identification of Y chromosome sequences in the human and Drosophila genomes. Genome Res. 23: 1894–1907. 23921660 10.1101/gr.156034.113 PMC3814889

[bib4] Charlesworth B. Charlesworth D., 1978 A model for the evolution of Dioecy and Gynodioecy. Am. Nat. 112: 975–997.

[bib5] Charlesworth D. Charlesworth B. Marais G., 2005 Steps in the evolution of heteromorphic sex chromosomes. Heredity 95: 118–128. 15931241 10.1038/sj.hdy.6800697

[bib6] Ellegren H., 2011 Sex-chromosome evolution: recent progress and the influence of male and female heterogamety. Nat. Rev. Genet. 12: 157–166. 21301475 10.1038/nrg2948

[bib7] Feschotte C. Pritham E. J., 2007 DNA transposons and the evolution of eukaryotic genomes. Annu. Rev. Genet. 41: 331–368. 18076328 10.1146/annurev.genet.40.110405.090448 PMC2167627

[bib8] Gregory T. R. Johnston J. S., 2008 Genome size diversity in the family Drosophilidae. Heredity 101: 228–238. 18523443 10.1038/hdy.2008.49

[bib9] Lahn B. T. Page D. C., 1999 Four evolutionary strata on the human X chromosome. Science 286: 964–967. 10542153 10.1126/science.286.5441.964

[bib10] Luo R. Liu B. Xie Y. Li Z. Huang W., 2012 SOAPdenovo2: an empirically improved memory-efficient short-read de novo assembler. GigaScience 1: 18. 23587118 10.1186/2047-217X-1-18 PMC3626529

[bib11] Mainx F., 1964 The genetics of Megaselia scalaris Loew (Phoridae): A new type of sex determination in Diptera. Am. Nat. 98: 415–430.

[bib12] Marçais G. Kingsford C., 2011 A fast, lock-free approach for efficient parallel counting of occurrences of k-mers. Bioinformatics 27: 764–770. 21217122 10.1093/bioinformatics/btr011 PMC3051319

[bib13] Mrackova M. Nicolas M. Hobza R. Negrutiu I. Monéger F., 2008 Independent origin of sex chromosomes in two species of the genus silene. Genetics 179: 1129–1133. 18558658 10.1534/genetics.107.085670 PMC2429867

[bib14] Rasmussen D. A. Noor M. A. F., 2009 What can you do with 0.1x genome coverage? A case study based on a genome survey of the scuttle fly Megaselia scalaris (Phoridae). BMC Genomics 10: 382. 19689807 10.1186/1471-2164-10-382 PMC2735751

[bib18] Traut W. Khuong N. t. Schneider S., 1990 Karyotypes of Megaselia scalaris (Diptera) wild-type and translocation strains. Genetica 83: 77–84.

[bib15] Traut W., 1994 Sex determination in the fly Megaselia scalaris, a model system for primary steps of sex chromosome evolution. Genetics 136: 1097–1104. 8005417 10.1093/genetics/136.3.1097 PMC1205866

[bib19] Traut W. Wollert B., 1998 An X/Y DNA segment from an early stage of sex chromosome differentiation in the fly Megaselia scalaris. Genome 41: 289–94. 9644837 10.1139/g98-015

[bib16] Traut W., 2010 New Y chromosomes and early stages of sex chromosome differentiation: sex determination in Megaselia. J. Genet. 89: 307–313. 20876997 10.1007/s12041-010-0042-x

[bib17] Willhoeft U. Traut W., 1990 Molecular differentiation of the homomorphic sex chromosomes in Megaselia scalaris (Diptera) detected by random DNA probes. Chromosoma 99: 237–242. 2209225 10.1007/BF01731698

