# Supplementary material for: How Big Is Your Y? A Genome Sequence-Based Estimate of the Size of the Male-Specific Region in Megaselia scalaris
Source: G3 (Bethesda). 2014 Nov 7;5(1):45–8. doi: 10.1534/g3.114.015057 (PMC4291468; doi:10.1534/g3.114.015057)
Supplement: Supporting Information [file supp_5_1_45__index.html]

How Big Is Your Y? A Genome Sequence-Based Estimate of the Size of the Male-Specific Region in Megaselia scalaris — Supporting Information 

# How Big Is Your Y? A Genome Sequence-Based Estimate of the Size of the Male-Specific Region in *Megaselia scalaris*

## Supporting Information for Hoehn and Noor, 2015

**Files in this Data Supplement:**

- Supporting Information - Table S1 and Figures S1-S3 (PDF, 350 KB)
- Table S1 - Assembly statistics from *Megaselia* male genome assembly: Output from SOAPdenoved2. (PDF, 97 KB)
- Figure S1 - Heatmap showing count of *Megaselia* 15-mers by log10(Contig Size) and percent of single copy 15-mers that were unmatched (USC) by female short read data. (PDF, 131 KB)
- Figure S2 - Estimates of male specific portion (MSP) of *M. scalaris* by minimum contig size and prior weight (a+b). (PDF, 197 KB)
- Figure S3 - Estimates of male specific portion (MSP) of *D. virilis* by minimum contig size and prior weight (a+b). (PDF, 178 KB)
