# Supplementary material for: How Big Is Your Y? A Genome Sequence-Based Estimate of the Size of the Male-Specific Region in Megaselia scalaris
Source: G3 (Bethesda). 2014 Nov 7;5(1):45–8. doi: 10.1534/g3.114.015057 (PMC4291468; doi:10.1534/g3.114.015057)
Supplement: Supporting Information [file supp_g3.114.015057_TableS1.pdf]

Table S1 Assembly statistics from *Megaselia* male genome assembly: Output from SOAPdenoved2.

Assembly Statistics: *Megaselia* Male

**Total Size (bp)**	453444693	
**Scaffold Number**	1755386	
**Mean Size**	258	
**Median Size**	183	
**Longest Sequence**	11221	
**Shortest Sequence**	100	
**Singleton Number**	1755386	
**Scaffolds > Length (bp)**	**Number**	**Percentage**
scaffolds>100	1743801	99.34%
scaffolds>500	156140	8.89%
scaffolds>1K	29777	1.70%
scaffolds>10K	2	0.00%
scaffolds>100K	0	0.00%
scaffolds>1M	0	0.00%
**Length Quantiles**	**Length**	**Number**
N10	978	31746
N20	643	90350
N30	476	173071
N40	368	281942
N50	292	420610
N60	236	593838
N70	193	806946
N80	160	1065221
N90	136	1373198
**Nucleotide Content**	**Number**	**Percentage**
Nucleotide A	153644768	33.88%
Nucleotide C	76208688	16.81%
Nucleotide G	74994329	16.54%
Nucleotide T	148596908	32.77%
GC Content	33.35%	
