# Supplementary material for: How Big Is Your Y? A Genome Sequence-Based Estimate of the Size of the Male-Specific Region in Megaselia scalaris
Source: G3 (Bethesda). 2014 Nov 7;5(1):45–8. doi: 10.1534/g3.114.015057 (PMC4291468; doi:10.1534/g3.114.015057)
Supplement: Supporting Information [file supp_g3.114.015057_FigureS1.pdf]

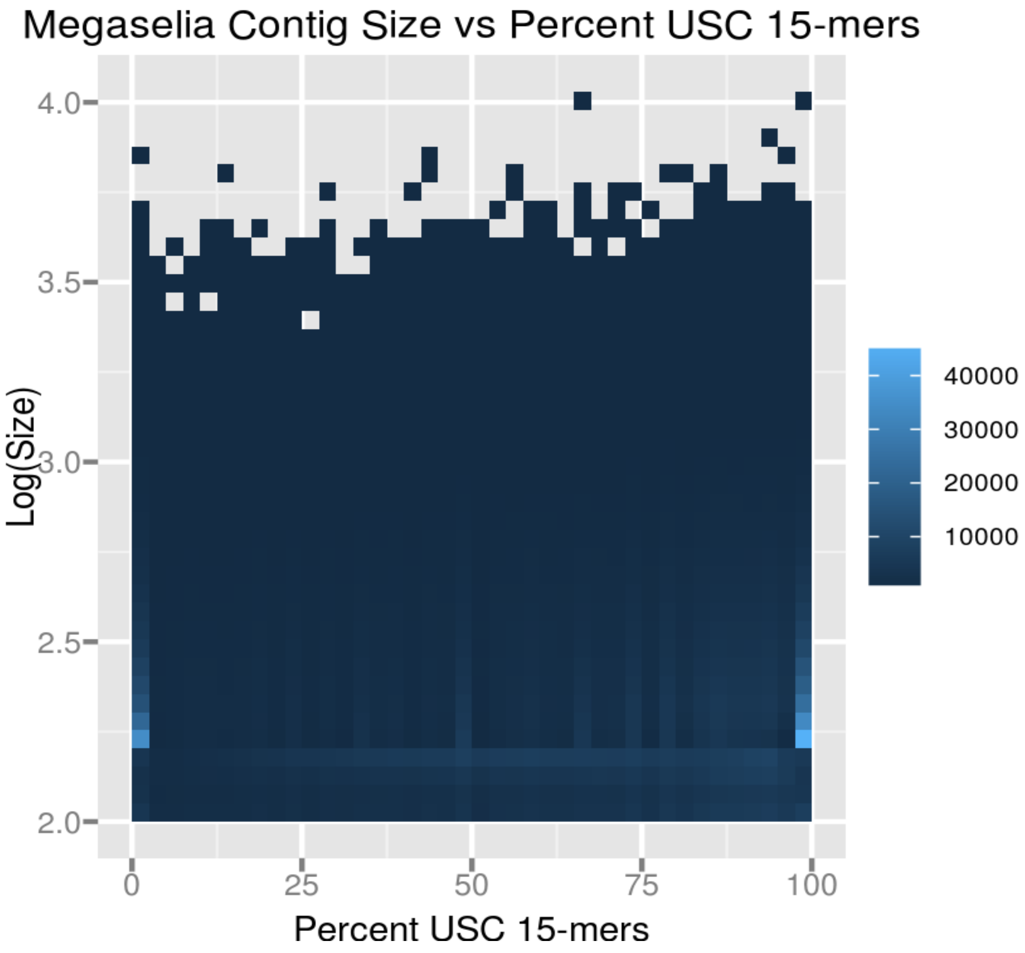

Figure S1 Heatmap showing count of *Megaselia* 15-mers by $\log_{10}$(Contig Size) and percent of single copy 15-mers that were unmatched (USC) by female short read data. Note peaks at 0% USC and 100% USC 15-mers. These are inferred to be caused by autosomal vs. male-specific contigs, respectively.
