# Supplementary material for: How Big Is Your Y? A Genome Sequence-Based Estimate of the Size of the Male-Specific Region in Megaselia scalaris
Source: G3 (Bethesda). 2014 Nov 7;5(1):45–8. doi: 10.1534/g3.114.015057 (PMC4291468; doi:10.1534/g3.114.015057)
Supplement: Supporting Information [file supp_g3.114.015057_FigureS3.pdf]

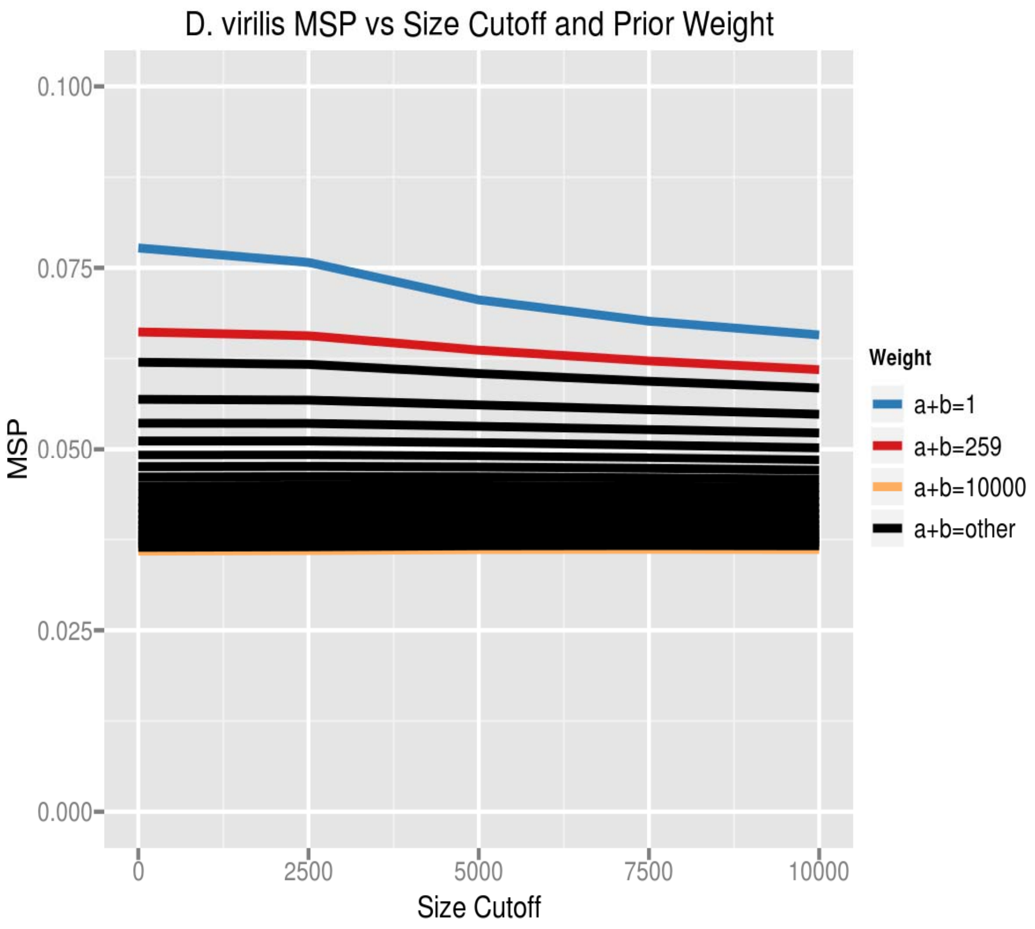

Figure S3 Estimates of male specific portion (MSP) of *D. virilis* by minimum contig size and prior weight (a+b). The least stringent estimate (a+b=1), median estimate (a+b=259), and most stringent estimate (a+b=10000) are colored. Prior weight values are otherwise selected in intervals of 500. These values are much higher than in the *Megaselia* estimation because the contigs of the *D. virilis* assembly are much larger (median contig size of 189 vs 4605, respectively).
